# *Pseudoscorpion Wolbachia* symbionts: diversity and evidence for a new supergroup S

**DOI:** 10.1186/s12866-020-01863-y

**Published:** 2020-06-30

**Authors:** Emilie Lefoulon, Travis Clark, Fanni Borveto, Marco Perriat-Sanguinet, Catherine Moulia, Barton E. Slatko, Laurent Gavotte

**Affiliations:** 1 Molecular Parasitology Group, Molecular Enzyme Division, New England Biolabs, Inc., Ipswich, USA; 2 School of Animal and Comparative Biomedical Sciences, University of Arizona, Tucson, USA; 3 ISEM, University of Montpellier, Montpellier, France

**Keywords:** *Wolbachia*, Pseudoscorpion, Symbiosis, Target enrichment, Genomics, Biotin

## Abstract

**Background:**

*Wolbachia* are the most widely spread endosymbiotic bacteria, present in a wide variety of insects and two families of nematodes. As of now, however, relatively little genomic data has been available. The *Wolbachia* symbiont can be parasitic, as described for many arthropod systems, an obligate mutualist, as in filarial nematodes or a combination of both in some organisms. They are currently classified into 16 monophyletic lineage groups (“supergroups”). Although the nature of these symbioses remains largely unknown, expanded *Wolbachia* genomic data will contribute to understanding their diverse symbiotic mechanisms and evolution.

**Results:**

This report focuses on *Wolbachia* infections in three pseudoscorpion species infected by two distinct groups of *Wolbachia* strains*,* based upon multi-locus phylogenies. *Geogarypus minor* harbours *w*Gmin and *Chthonius ischnocheles* harbours *w*Cisc, both closely related to supergroup H, while *Atemnus politus* harbours *w*Apol, a member of a novel supergroup S along with *Wolbachia* from the pseudoscorpion *Cordylochernes scorpioides* (*w*Csco). *Wolbachia* supergroup S is most closely related to *Wolbachia* supergroups C and F. Using target enrichment by hybridization with *Wolbachia*-specific biotinylated probes to capture large fragments of *Wolbachia* DNA, we produced two draft genomes of *w*Apol. Annotation of *w*Apol highlights presence of a biotin operon, which is incomplete in many sequenced *Wolbachia* genomes.

**Conclusions:**

The present study highlights at least two symbiont acquisition events among pseudoscorpion species. Phylogenomic analysis indicates that the *Wolbachia* from *Atemnus politus* (*w*Apol), forms a separate supergroup (“S”) with the *Wolbachia* from *Cordylochernes scorpioides (w*Csco). Interestingly, the biotin operon, present in *w*Apol, appears to have been horizontally transferred multiple times along *Wolbachia* evolutionary history.

## Background

*Wolbachia* are endosymbiotic alpha-proteobacteria infecting a broad range of arthropods and nematodes [1, 2]. The bacteria of this genus are considered to be the most widely spread symbionts in the animal world, perhaps infecting half of insect species [3–5]. *Wolbachia* are maternally inherited and can induce variable phenotypes in their hosts through mutualism or parasitism [6–9]. *Wolbachia* are genetically diverse, as are the interactions with their hosts [10, 11]. Currently, there is a general consensus to classify them in monophyletic lineage groups (“supergroups” A to R). *Wolbachia* belonging to supergroups C, D and J exclusively infect filarial nematodes (Onchocercidae) [12–14]. Supergroup L exclusively contains plant parasitic nematodes (Pratylenchidae) [15, 16]. Supergroup F *Wolbachia* is, so far, the only clade composed by some strains infecting arthropods and some infecting filarial nematodes [17, 18]. All other described supergroups exclusively infect arthropods [19–23]. The proposed supergroup G members [24] and R [25] are now considered to be part of supergroup B [26] and A [27], respectively, and are no longer considered as separate supergroups.

In the last few years, the number of published *Wolbachia* genomes has increased and currently 48 draft and 21 complete genomes of *Wolbachia* are available at the NCBI database. However, these data are not a good representation of the entirety of *Wolbachia* diversity. Indeed, among the 21 complete genomes, 15 are symbionts of insects belonging to either supergroup A or B (including 7 strains from species of *Drosophila*), 2 are symbionts of insects belonging to other supergroups (F and E) and 4 are symbionts of nematodes (supergroups L, C or D). *Wolbachia* has been identified in other arthropods, such as isopods [28, 29] and arachnids [24]. Limited genomic data for isopods are available, for example a draft genome of *w*Con, infecting *Cylisticus convexus* and a draft genome of *w*VulC infecting *Armadillidium vulgare* [30]. No genomic data is available for arachnid symbionts while several studies identified *Wolbachia* in spiders [24, 25, 31], mites [20] or scorpions [32].

In the current study, we focused on the *Wolbachia* symbionts of pseudoscorpions. Pseudoscorpions inhabit wooded areas associated with rotting vegetation. Unlike most arthropods, they are viviparous with embryos obtaining nutrients from the maternal reproductive tract [33]. The presence of *Wolbachia* was described for the first time in pseudoscorpions in 2005 in *Cordylochernes scorpioides* [34]. It has been reported that the endosymbiont is responsible for the “male killing” phenotype in *C. scorpioides*, associated with a high rate of spontaneous abortion.

We examined the prevalence of *Wolbachia* infections in pseudoscorpion population samples from an area in Montpellier (France) where three different species of pseudoscorpions were collected and determined to be positive for *Wolbachia* infection. The molecular analyses phylogenetically identified two different *Wolbachia* groups, of which one strain is divergent from currently accepted *Wolbachia* supergroups. We used *Wolbachia* DNA enrichment capture [35, 36] to ensnare *Wolbachia* DNA for genomic sequencing from this *Wolbachia* strain, *wApo,* infecting *Atemnus politus*.

## Results

### Identification and evolution of Pseudoscorpions

**Fig. 1:**
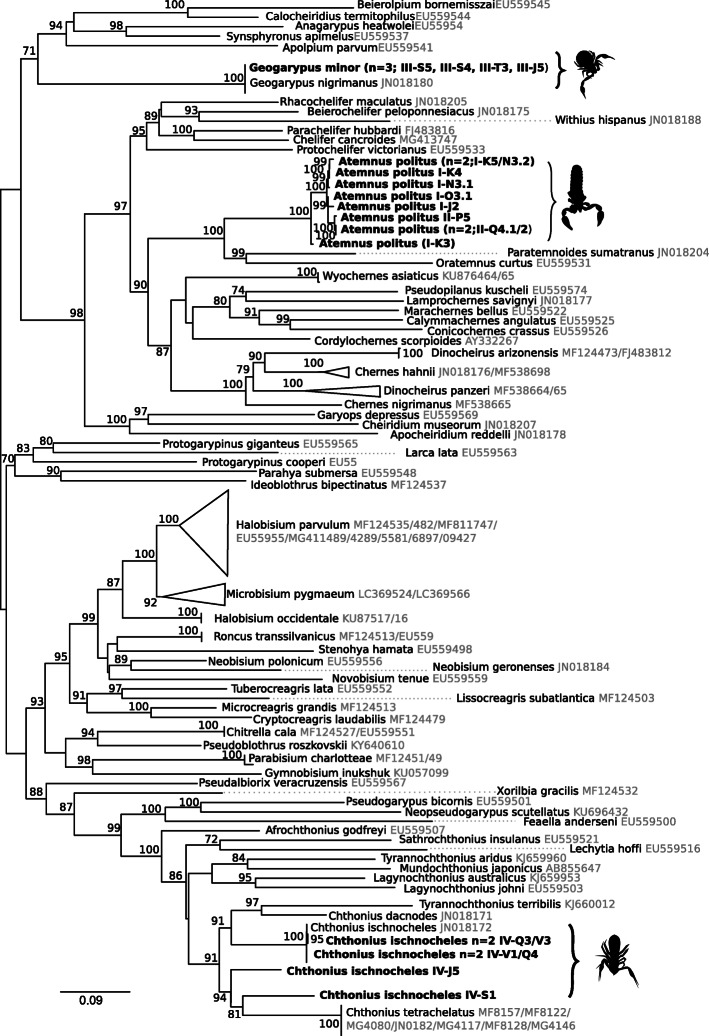
Phylogenetic tree of pseudoscorpions based on the COI (mitochondrial cytochrome oxidase I) gene. The total length of datasets is 659 bp. The topology was inferred using Maximum Likelihood (ML) inference using IQTREE [39]. The Best-fit model calculated using ModelFinder according to BIC index was TVM + R7. Nodes are associated with Bootstrap values based on 1000 replicates, only bootstrap values superior to 70 are indicated

In all, 94 pseudoscorpion specimens were collected from an area of Montpellier (France). Sixty specimens were morphologically identified as *Atemnus politus* (Simon, 1878), 24 specimens belong to the species *Geogarypus minor* (Koch, 1873) and 10 specimens belong to the species *Chthonius ischnocheles* (Hermann, 1804). The specimen identification is consistent with the analysis of the mitochondrial cytochrome oxidase I gene marker (*COI*). The COI sequences of the specimens belonging to *Geogarypus minor* are identical to each other and to the sequence of *Geogarypus nigrimanus* (JN018180 specimen voucher MNHN-JAB62). The species *Geogarypus nigrimanus* has been recently synonymized with *Geogarypus minor* [37]. Regarding the produced COI sequences of the specimens belonging to *Chthonius ischnocheles* species, the specimens IV3–1, Q3–1, IV1–1 and Q4 are between 99.8–100% identical and match the sequence of *Chthonius ischnocheles* available at the NCBI database (JN018172 specimen voucher MNHN-JAB62). The existence of cryptic species of *Chthonius ischnocheles* has been documented [38] and the specimens IV-J5 and IV-S1 form a paraphyletic group with the other *Chthonius ischnocheles* (Fig. 1).

The COI sequences of the specimens identified as *Atemnus politus* present more intraspecific variability: calculated pairwise similarities range between 97.7–99.8%. The blastn analysis [40] does not provide identification of strongly similar sequences in databases; indeed no COI sequences from species related to the *Atemnus* genus have been deposited in the NCBI database. The most similar COI sequence in the database belongs to *Atemnidae* sp. JA-2011 voucher (JN018203), showing only 79.74% similarity. The COI-based phylogeny indicates that all the specimens of *Atemnus politus* form a clade which is a sister group of the clade composed of *Paratemnoides sumatranus* and *Oratemnus curtus* species, both representatives of the *Atemnidae* family (Fig. 1). The COI analysis shows all specimens of *Chthonius ischnocheles* form a sister group of other species belonging to *Chthonius* species (*C. tetrachelatus*, *C. dacnodes*) (Fig. 1). To date, the only species of pseudoscorpion described as a *Wolbachia* host, *Cordylochernes scorpioides*, belongs to the Chernetinae and is a sister group to the Atemnidae family (including *Atemnus politus*) (Fig. 1).

### Prevalence of *Wolbachia* infection

*Wolbachia* was detected by PCR amplification of *wsp* and *gatB* markers (see Methods) in the three studied pseudoscorpion species and in accordance with the generally used nomenclature of *Wolbachia* strains, we have named these new strains according to their hosts: *w*Apol for bacteria infecting *Atemnus politus*, *w*Gmin for bacteria infecting *Geogarypus minor* and *w*Cisc for bacteria infecting *Chthonius ischnocheles*.

The *A. politus* specimens have the highest prevalence of *Wolbachia* infection with 43.3% positive samples (26 positive of 60 specimens examined). 16.7% of *G. minor* individuals appear to be infected (4 positive of 24 specimens examined), while only 10% of the *C. ischnocheles* (1 positive of 10 specimens examined) show PCR amplification of the *Wolbachia* markers (Table S1).

### The *w*Apol draft genome and annotation

Two draft genomes were produced for *w*Apol *Wolbachia* from *Atemnus politus* (specimens K5 and K3) using target capture enrichment [35, 36]. For Illumina sequencing, an enrichment of 45-fold was observed (0.4% of the reads mapped to the draft genome without enrichment against 18% with) and 50-fold enrichment was observed with PacBio sequencing (0.8% of the reads mapped to the draft genome without enrichment as opposed to 40.9% with). Unfortunately, the amounts of DNA available for the two specimens were low, and thus the samples were processed simultaneously using individual barcoded adaptors (see Methods) and for PacBio sequencing, relatively few total reads were produced (Table 1). The hydrid de novo assembly allowed production of two draft genomes. For specimen K5, a 373 contig draft genome of 1,445,964 bp was obtained, with an average G + C content of 35.6% (largest contig, 25,286 bp, N50 = 5741 bp, average sequencing depth of 420X). For specimen K3, a 200 contig draft genome of 1,404,177 bp was obtained, with an average G + C content of 35.49% (largest contig, 40,755 bp, N50 = 10,346 bp, average sequencing depth = 205X) (Table 1).

**Table 1:** Information for the *w*Apol genome sequences

		*w*Apol K5	*w*Apol K3
Before assembly	Illumina reads without enrichment	76,487,892	8,349,854
	Illumina reads with enrichment	8,714,832	7,422,205
	CCS PacBio reads without enrichment	13,467	2091
	CCS PacBio reads with enrichment	2032	13,070
Selection of reads mapped to the assembly	Illumina reads without enrichment	109,309 (0.4%)	166,537 (1.99%)
	Illumina reads with enrichment	1,912,222 (18%)	1,428,179 (19.24%)
	CCS PacBio reads without enrichment	110 (0.8%)	35 (1.6%)
	CCS PacBio reads with enrichment	885 (40.9%)	3343 (25.57%)
Draft genome assembly	Number of contigs	373	200
	Size of the largest contig	25,286	40,755
	Total length (bp)	1,445,964	1,404,177
	Contigs > = 10,000 bp	28	43
	N50	5741	10,346
	L50	73	40
	GC%	35.61	35.49
	Number of Coding Sequences	1746	2215
	Number of RNAs	39	45
BUSCO analysis	Complete BUSCOs %	70.2%	69%
	Fragmented BUSCOs %	7.2%	8.2%
	Missing BUSCOs %	22.6%	22%

Summary of de novo assembly processed in the current study using Unicycler: statistics using QUAST [41]; annotation using RAST [42] pipeline; assessment of the draft using BUSCO v3 [43]

Among 221 single-copy orthologue genes conserved among proteobacteria (BUSCOs database), 155 are present in the *w*Apol K5 draft genome and 151 in *w*Apol K3, suggesting 70.2 and 69% complete BUSCOs, respectively. This percentage can be used to assess the level of completeness of genomes (see Methods). By comparison, the *Wolbachia* from *Drosophila melanogaster, w*Mel*,* has a higher level of BUSCOs in the current analysis with 180 genes (81.4%) and *Wolbachia* from *Pratylenchus penetrans, w*Ppe, has a low level with 161 genes (72.9%) (Table S2). The low level of BUSCOs observed for *w*Apol suggest that either the *w*Apol draft is incomplete or this genome is highly degraded as has been described for *Wolbachia* from *Onchocerca ochengi* (74.7% complete BUSCOs) [44].

**Fig. 2:**
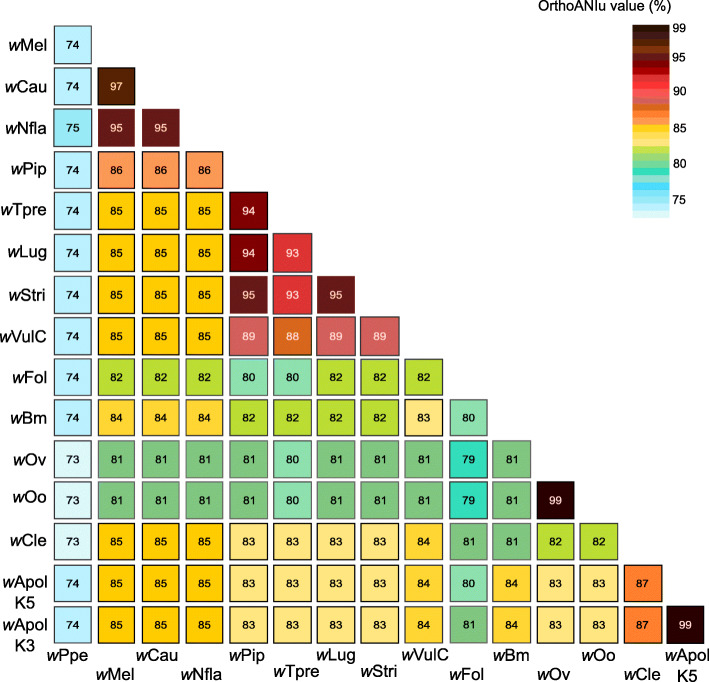
Summary of ANI calculations for *Wolbachia* genomes. The Average Nucleotide Identity (ANI) between the wApol draft genomes and 13 complete genomes of *Wolbachia* evaluated using the ANI Calculator [45]

The Average Nucleotide Identity (ANI) calculation (see Methods) indicates that the two *w*Apol drafts have 99% identity and they are most similar to *w*Cle with 87% identity (Fig. 2). According to the analysis of available complete genomes, strains which are representative of the same supergroup share more than 94% identity; for example, 99% for *w*Oo and *w*Ov (from supergroup C), 94% for *w*Pip and *w*Tpre (from supergroup B) or 96% for *w*Mel and *w*Cau (from supergroup A).

One thousand seven hundred forty-six coding sequences (CDS) were annotated using RAST from *w*Apol K5 and 2215 CDS from *w*Apol K3. The high number of CDSs might be explained by presence of multiple copies of same genes due to incompleteness of the draft genomes. The two draft genomes contain a low number of transposable elements (55 mobile elements, 16 group II intron-associated genes, 52 ISs for K5; 35 mobile elements, 9 group II intron-associated genes, 36 ISs for K3) compared to other *Wolbachia* strains infecting insects (with the exception of *w*Tpre), but a high number compared to *Wolbachia* infecting filarial nematodes (Table S3). Numerous phage-like genes were observed in the *w*Apol K5 draft genome, with 54 CDS (coding sequences) detected on 37 different contigs with 9 annotated as prophage sequences (Table S4). Fewer phage-like genes were detected in the *w*Apol K3 draft genome, with 20 CDS detected on 12 different contigs and only one annoted as a prophage sequence (Table S4). The number of phage-like genes is highly variable among the studied *Wolbachia* genomes and the number observed in *w*Apol is close to *w*Mel (with 44 CDS) or *w*Cle (with 20 CDS). However, the variability in transposable element and phage numbers observed between the two *w*Apol draft genomes suggests that the genome sequences are incomplete.

**Fig. 3:**
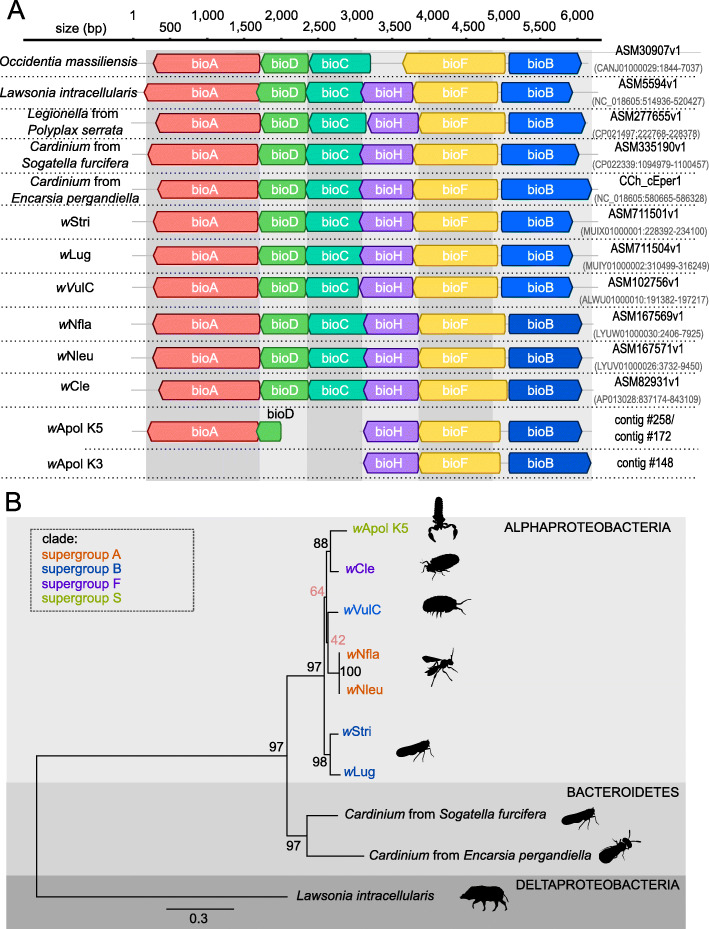
Organization and molecular analysis of the biotin operons of *Wolbachia*. **a** Organization of the biotin operon. The operons among the *Wolbachia* and four outgroups were identified and then aligned using MAFFT [46]. **b** Phylogeny of the biotin operon (concatenation of contig containing bioA and bioD with the contig containing bioH, bioF and bioB). The length of datasets is 4603 bp. The topology was inferred using Maximum Likelihood (ML) inference using IQ-TREE [39]. The Best-fit model calculated using ModelFinder according to AIC index was TVM + I + G4. Nodes are associated with Bootstrap values based on 1000 replicates. The *Wolbachia* supergroups are indicated by the color: purple for supergroup F; dark blue for supergroup A; orange for supergroup A and green for supergroup S

Although the *w*Apol drafts are likely not complete, functional annotation of genes by KAAS highlights that the biotin pathway is present, while it is absent in most other studied *Wolbachia* (Table S5). Only *w*Cle (supergroup F), *w*Nfla, wNleu (supergroup A), *w*Lug, *w*Stri and *w*VulC (supergroup B) have a complete biotin pathway. Only *bioC* is not detected in the *w*Apol K5 draft, but the operon is detected on two contigs: the genes bioA and bioD on contig #258 and the genes bioH, bioF and bioB on contig #172 (Fig. 3a). In all studied *Wolbachia* which contain the operon, the bioC gene is present between the *bioD* and *bioH* genes and the absence might be due to the fragmentation of the *w*Apol K5 draft sequence (Fig. 3a). For the *w*Apol K3 draft genome, only one contig (#148) containing *bioH*, *bioF* and *bioB* was detected. The organization of the biotin operon appears to be conserved between all *Wolbachia* genomes which contain the complete pathway (Fig. 3a). The phylogenies of the biotin genes (containing bioA, bioB, bioH, bioD and bioF) shows that the biotin operon of wApol K5 is most closely related to the operon of wCle (Fig. 3b).

### Multi-locus phylogenies and phylogenomics

**Fig. 4:**
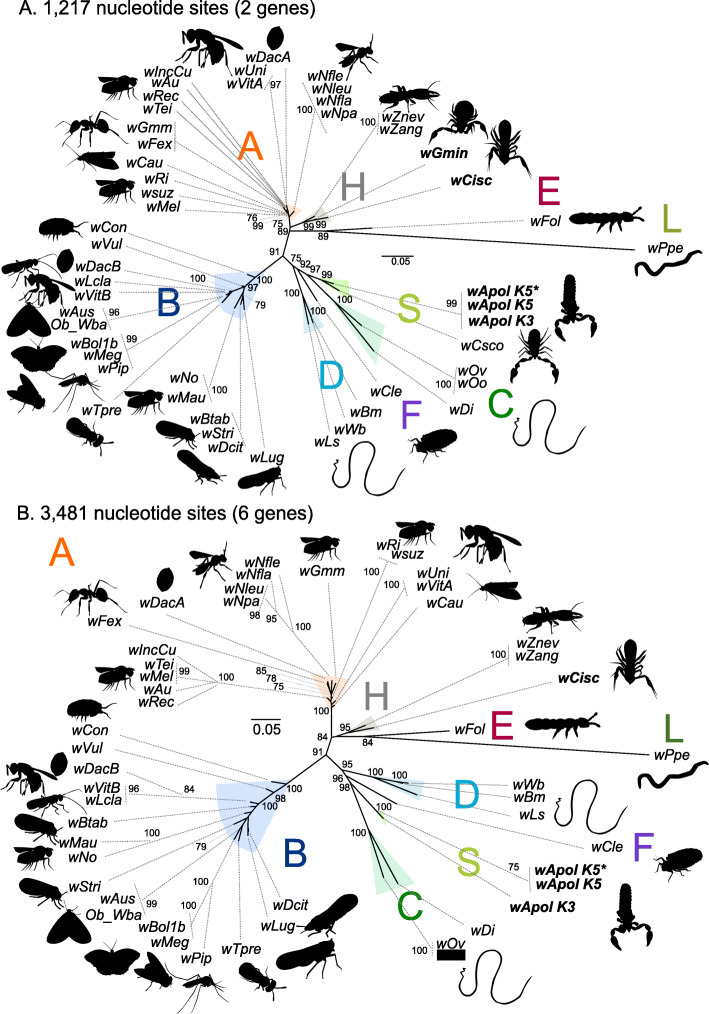
Unrooted phylogenetic trees of *Wolbachia* based on 2 and 6 markers by Maximum Likelihood. **a** Analysis based on concatenation of *ftsZ* and *coxA*; the total length of datasets is 1217 bp. The topology was inferred using Maximum Likelihood (ML) inference using IQ-TREE [39]. The Best-fit model calculated using ModelFinder according to BIC index was TIM3 + I + G4. **b** Analysis based on concatenation of 16S rDNA, *dnaA*, *ftsZ*, *coxA*, *fbpA* and *gatB*; the total length of datasets is 3481 bp. The Best-fit model calculated using ModelFinder according to BIC index was GTR + I + G4. Nodes are associated with Bootstrap values based on 1000 replicates, only bootstrap value superior to 70 are indicated. The *Wolbachia* supergroups (A–S) are indicated. The bolded names indicate data produced in this study and in the case of wApol, the asterisk (*) indicates sequences produced by PCR amplification

The analysis of *Wolbachia* in *Geogarypus minor, w*Gmin, was more complex. Two genes were sequenced for *w*Gmin, *coxA* and *ftsZ*, and only nested PCR provided clean sequences to enable phylogenetic comparisons. The *Wolbachia* phylogeny, based on the two genes, identified two different sub-groups of *Wolbachia* in our samples (Fig. 4a). The *Wolbachia* infecting *Chthonius ischnocheles, w*Cisc, and *Geogarypus minor*, *w*Gmin, group together and appear closely related to supergoup H *Wolbachia* infecting *Zootermopsis* termite species (*w*Zneg and *w*Zang). The *Wolbachia* from *Atemnus politus, w*Apol, and the *Wolbachia* from *Cordylochernes scorpioides*, *w*Csco, form a clade which is divergent from known supergroups. This clade is a sister group to supergroup C *Wolbachia,* which contains the symbiont present in filarial nematodes (*Onchocerca* spp. and *Dirofilaria immitis*). The phylogeny based on six genes (16S rDNA, *ftsZ*, *dnaA*, *gatB*, *fbpA* and *coxA*) confirms the topology of *w*Apol and *w*Cisc (*w*Gmin and *w*Csco not included) (Fig. 4b). Our analysis shows the same topology for *w*Apol sequences from the two draft genomes (K5 and K3) as well as sequences of *w*Apol K5 amplified by PCR, confirming that this topology is not an artifact of draft missassembly. In addition, to verify the accuracy of the *Wolbachia* presence in *Geogarypus minor*, the *coxA* gene was sequenced from another specimen (III-J5) derived from an independent DNA extraction, and PCR amplification and the sequences were identical to another specimen III-T3 (accession number MT273088).

The *Wolbachia* from *Cordylochernes scorpioides* had been previously studied using a set of genes not routinely sequenced for *Wolbachia* evolutionary studies (*groEL*, *fabK*, *nuoG*, *NADH dehydrogenase I subunit F*, *aspS*, *gltA*, *coxA*, *ftsZ*, *wsp*, *orpB*, *nuoD*, isocitrate dehydrogenase gene, TPR domain-containing protein gene) [47]. We performed a multi-locus phylogeny based on these 13 genes, including *w*Csco and sequences isolated from available complete or draft genomes of *Wolbachia*, as well as those from the *w*Apol produced draft genomes. The observed phylogeny shows that *w*Csco and *w*Apol cluster together to form a separate clade, a closely related sister group to supergroup C, as observed with the phylogeny based on only two markers (Fig. 4a). The two pseudoscorpions contain closely related *Wolbachia* strains, with 89.3% identity among these 13 genetic markers (Figure S1).

**Fig. 5:**
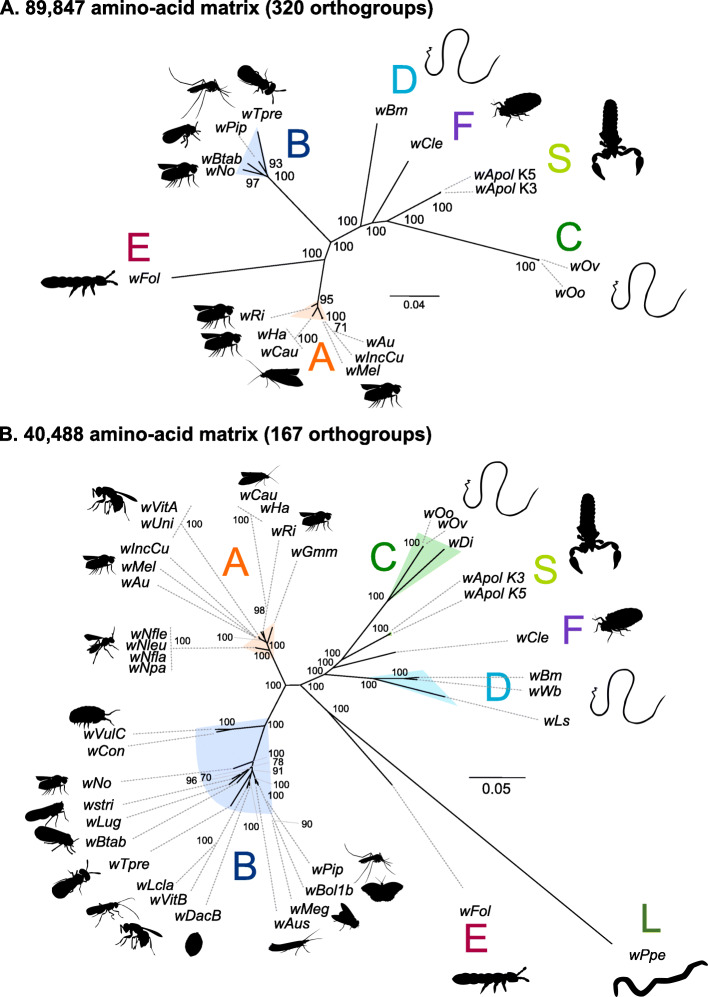
Phylogenomic analyses of *Wolbachia*. **a** Analysis based on concatenation of 320 single-copy orthogroups representing an 89,847 amino-acid matrix. The topology was inferred using Maximum Likelihood (ML) inference using IQTREE [39]. The Best-fit model calculated using ModelFinder according to BIC index was JTT + F + I + G4. **b** Analysis based on concatenation of 167 single-copy orthogroups representing 40,488 amino-acid matrix. The Best-fit model calculated using ModelFinder according to BIC index was JTT + F + I + G4. Nodes are associated with Bootstrap values based on 1000 replicates, only bootstrap value superior to 70 are indicated. The *Wolbachia* supergroups (A–S) are indicated

Two complete phylogenomic analyses were performed: one including 17 *Wolbachia* genomes representing 6 different supergroups and one including 38 *Wolbachia* genomes representing 7 different supergroups and including additional draft genome sequences. Three hundred twenty single-copy orthologues were identified among the 17 *Wolbachia* genomes, while 166 were identified among the 38 *Wolbachia* genomes. The Maximum Likelihood phylogeny based on the reduced matrix (17 genomes, 320 orthologues, 89,847 amino-acid sites) (Fig. 5a) and the one based on the larger matrix (38 *Wolbachia*, 167 orthologues, 40,488 amino-acid sites) (Fig. 5b) indicates the *w*Apol group does not cluster with any other *Wolbachia* group, suggesting it evolved from an independent separate speciation event. We created supplementary phylogenies based on 16S rRNA and *ftsZ* and have included numerous *Wolbachia* species to be sure that *w*Apol and *w*Csco are not grouped with previously described *Wolbachia* supergroups (Figure S2 and S3).

Based upon the phylogenomics analyses, *w*Apol (supergroup S) is closely related to supergroup C (symbionts within the filarial nematodes *Onchocerca* spp. and *Dirofilaria immitis*) and supergroup F (including both filarial and arthropods symbionts).

## Discussion

### Diversity of *Wolbachia* in pseudoscorpions

This study highlights diversity of *Wolbachia* harboured by pseudoscorpions. The studied specimens harbouring distinct *Wolbachia* represent different families, not closely related, within pseudoscorpions. The small number of pseudoscorpion species studied (3 among 3300 described species) represent only small snapshot of the diversity within this group (3 families among 27 described), suggesting that the heterogeneity of *Wolbachia* among pseudoscorpion species may likely be an underestimate. The species *Geogarypus minor*, a representative of the Geogarypidae family and the species *Chthonius ischnocheles*, a representative of the Chthoniidae family, contain *Wolbachia* closely related to each other and related to supergroup H, as represented by termite *Wolbachia*. The species *Atemnus politus*, as a representative of the Atemnidae family, contains *Wolbachia* closely related to the *Wolbachia* from the previously described pseudoscorpion *Cordylochernes scorpioides* and forms a clade as a sister group to supergroup C (infecting exclusively filarial nematodes) and supergroup F (infecting filarial nematodes and arthropods). Within the evolutionary history of pseudoscorpions, at least two events of horizontal transfers of *Wolbachia* infection have apparantly occurred. More detailed, biodiversity analysis of this group will be required to fully detail the evolutionary dynamics of their *Wolbachia*.

### New supergroup S

The *Wolbachia* harboured by *Atemnus politus* (*w*Apol) and *Cordylochernes scorpioides* (*w*Csco) form a clade divergent from those *Wolbachia* supergroups previously described (Figure S2 and S3). The analysis of the *w*Apol draft genome using phylogenomic analysis (Fig. 5), or ANI value (Fig. 2) point out a divergent *Wolbachia* supergroup. It is important to distinguish the notion of “supergroup” from the notion of “species” (which remains problematic for bacteria [48]). The “supergroup” naming only describes the different evolutionary lineages (or clades) of *Wolbachia,* and their limits remain arbitrary (as far as it being a monophyletic group).

In the past, two W*olbachia* supergroups had been designated and then revised based on further phylogenetic analysis. Supergoup G had been described as a clade of *Wolbachia* from spider *Diaea* species based on *wsp* marker analysis [49]. However, it was later demonstrated that the analysis based upon this marker sequence led to an incorrect conclusion due to recombination events between *Wolbachia* from supergroups A and B [26]. In the case of supergroup R, four genes (*ftsZ*, *coxA*, *groEL*, and 16S) were analyzed to study *Wolbachia* from cave spiders (*Telema* spp.) [25]. From among these four genes, only the *groEL* marker gave a different topology, excluding these *Wolbachia* from supergroup A. Upon reanalysis, the differing topology conclusion appears to be due to a G + C composition bias of the *groEL* gene, causing an incorrect phylogenetic analysis when based on nucleotide sequences [27]. Being aware of these, we created supplementary phylogenies based on 16S rRNA and *ftsZ* and have included numerous *Wolbachia* species to be sure that *w*Apol and *w*Csco are not grouped with previously described *Wolbachia* supergroups (Figure S2 and S3).

To ensure that the draft genomes of *w*Apol did not create an artificial clade, we further sequenced PCR amplificons from six previously studied marker genes from an independent specimen from the same set of samples as K5 and verified that the data provided the same topology. Based upon this confirmation, we propose that *Wolbachia* from *Atemnus politus* and *Cordylochernes scorpioides* constitute representatives of a new supergroup. To avoid any confusion, we propose not to reuse the previous supergroup R [27] designation and have assigned *Wolbachia* from the pseudoscorpion *Atemnus politus* and *Cordylochernes scorpioides* to a novel supergroup S.

### *w*Apol draft genomes and limitation of de novo assembly based on the enrichment method

Two draft genomes, *w*Apol K3 (1,404,177 bp long) and *w*Apol K5 (1,445,964 bp long) were produced, as representatives of supergroup S. These represent an average size for a *Wolbachia* genome from arthropods with the smallest complete *Wolbachia* genome being the symbiont from *Trichogramma pretiosum* (*w*Tpre) which is 1,133,809 bp long [50] and the largest being the symbiont from *Folsomia candida* (*w*Fol) which is 1,801,626 bp long [11]. The genomes of *Wolbachia* from filarial nematodes are smaller, between 957,990 bp and 1,080,084 bp [44, 51]. However, the two *w*Apol draft genomes are fragmented, being in 200 contigs and 373 contigs, respectively. Relatively few PacBio reads were obtained due to limited material. Despite an efficient probe hybridization enrichment, the low DNA input required pooling of barcoded samples. Based upon BUSCO, assessment of genome assembly indicates that the draft genomes *w*Apol are probably incomplete. Despite its average size, the percentage of missing single-copy orthologs conserved among proteobacteria and *w*Apol (or missing BUSCOs) (around 22% for K3 and 22.6% for K5) is close to the percentage observed for smaller genomes as *w*Oo (22.6%; 957,990 bp), *w*Ov (23.1; 960,618 bp) or *w*Ppe (21.7%; 975,127 bp). Further, the frequency of transposable elements (Group II intron-associated genes, mobile elements and IS) varies between the two drafts and lower than usually observed for *Wolbachia* from arthropods (Table S3). In addition, the long repetitive elements in a genome can limit the efficiency of de novo assembly following hybridization capture protocols, as shown with the de novo assembly of *w*AlbB (*Wolbachia* from *Aedes albopictus*), which contains a large numbers of long repetitive Group II intron-associated genes [36].

Since the first observation in *Wolbachia* from *Culex pipiens*, the presence of WO bacteriophage in *Wolbachia* has been well documented [52–55]. Not all *Wolbachia* genomes have intact prophage regions; only vestiges of prophage DNA were detected in *Wolbachia* genomes infecting nematodes belonging to D or L supergoups [15, 44, 51]. In the two *w*Apol draft genomes, we identified several coding sequences annoted as phage-like proteins, such as structural phage proteins (tail, portal phage, capsid genes) (Table S4). Although, the fragmented nature of the draft genomes limits interpretation, *w*Apol has clearly been associated with WO bacteriophage, unlike supergroup C *Wolbachia* in which no structural phage proteins are found (Table S4) [44]. In addition, the presence of a prophage region appears to be a limitation for de novo genome assembly using the *Wolbachia* genome enrichment method, perhaps due to missing probes based upon the original design or the repetitive pattern of this region, a similar observation to that of the *w*AlbB genome assembly, which also failed to assemble the WO region [36].

### Rare presence of biotin pathway in *Wolbachia*

Although it is difficult to conclude about the absence of a particular gene because the *w*Apol draft genomes are likely incomplete, our study highlights the biotin pathway as being more complete in *w*Apol than in numerous studied *Wolbachia*. Until recently, only *w*Cle (*Wolbachia* from bedbug *Cimex lectularius*) contains a complete pathway for biotin (vitamin B7) [56]. More recently, a complete pathway for biotin was identified in *w*Nfla and *w*Nleu (supergroup A) [57], as well as in *w*Lug and *w*Stri (supergroup B) [58]. Interestingly, *w*Cle provisions biotin (but not thiamin), which significantly contributes to the fitness of host bedbug [56]. In planthoppers, *w*Lug and *w*Stri *Wolbachia* appear to increase the fecundity of their hosts, which may be related to a beneficial effect of *Wolbachia*-synthesized biotin and riboflavin [58]. It is tempting to speculate that this pathway in *w*Apol may also be important in the association between *Wolbachia* and its pseudoscorpion hosts. From this analysis, we also identified the complete biotin pathway for *Wolbachia* from the isopod *Armadillidium vulgare w*VulC genome. The presence of the biotin operon among the *Wolbachia* appears rare, but when present, its physical organization appears conserved among them (Fig. 3). Phylogenetic analysis of the biotin gene cluster supports the suggestion that the biotin operons were acquired by lateral transfer from endosymbiotic bacteria, such as *Cardinium* species, as previously suggested [57, 58]. The lack of congruence between the phylogeny of the biotin operons and *Wolbachia* phylogeny suggests multiple independent transfers of the operon within *Wolbachia* evolutionary history.

## Conclusion

Our results emphasize the diversity of *Wolbachia* among the pseudoscorpion family. We identified infection with two different groups of *Wolbachia,* suggesting their independent evolutionary inheritance*,* likely via host-switching. Horizontal transmission of *Wolbachia* among insects has been previously documented [31, 59, 60] but this is the first time that a group of pseudoscorpions has been similarly analyzed. The *Wolbachia* symbiont from *Atemnus politus, w*Apol is divergent from previous described supergroups and we propose a novel clade composed of *w*Apol and *w*Csco, named supergroup S. The multi-locus phylogenies and phylogenomics indicate that the supergroup S is closely related to the supergroup F (which contains *Wolbachia* symbionts of arthropods and filarial nematodes) and supergroup C (comprised exclusively *Wolbachia* of filarial nematodes).

We produced two draft genomes of *w*Apol, one of 200 contigs (1,404,177 bp; K3) and the second of 373 contigs (1,445,964 bp; K5) using target enrichment capture. The genome sequences remain incomplete due to the low input of DNA, limiting the PacBio long read sequencing and due to the presence of large repetitive motifs and prophage region(s), previously demonstrated to interfere with complete genome assembly based on the *Wolbachia* genome capture enrichment.

Interestingly, the annotation of the *w*Apol draft genome contains the biotin pathway, as opposed to its lack in most of other *Wolbachia*, while speculative, it might be suggested that *w*Apol may provision biotin to their pseudoscorpion hosts, similar to *w*Cle in its bedbug host or *w*Stri and *w*Lug in planthoppers. The nature of the pseudoscorpion-*Wolbachia* biology emphasizes the amazing complexity and evolutionary trajectories of these ubiquitous symbionts and provides the background for future comparative studies.

## Methods

### Source of material, DNA extraction and characterization

Specimens were caught in the field using classical soil microfauna recovery methods using a Berlese–Tullgren funnel [49, 61]. The specimens were collected at Montpellier in 2017 (43°37′52.0″N 3°52′04.6″E).

DNA from specimens was extracted using the Monarch® Genomic DNA Purification Kit following the recommended protocol for extraction from tissues (New England Biolabs (NEB), USA) with overnight incubation at 56 °C with proteinase K. The quality of the extraction was verified by a PCR amplification of the host COI gene (Table S6). A total of 20 sequences were deposited in the GenBank Data Library: MN923050-MN923069 (Table S7).

### Detection and molecular characterization of *Wolbachia* symbionts

The determination of *Wolbachia* infection in populations was determined by a series of individual specific PCRs (Table S1). The presence of *Wolbachia* was initially screened using PCR amplification of the *Wolbachia* surface protein (*wsp*) gene. The presence of *Wolbachia* was further confirmed using PCR amplification of the *gatB* gene for all specimens which tested positive for *wsp* amplification or for those giving dubious results, as well as a randomly selected set of specimens which were negative for *wsp* amplification.

The molecular characterization of *Wolbachia* was determined by PCR amplification of six genes (16S rDNA gene, *dnaA*, *coxA*, *fbpA*, *gatB* and *ftsZ*) as described in Lefoulon et al. [14] (Table S6). For some specimens, nested PCR amplification was necessary to obtain sufficient PCR product. A total of 14 sequences were deposited in the GenBank Data Library: MN931247 to MN931248, MN931689 to MN931700 and MT273088 (Table S8).

### Next-generation sequencing of *Atemnus politus*

*Atemnus politus* specimens (K5 and K3) were collected from the same location, but at different times within the same year (respectively 5 April and 22 February 2017). The extracted DNAs were low (around 350 ng total based on Nanodrop™ quantification Thermo Scientific, USA)) and fragmented (average size around 6000 bp). In order to attain an amount of DNA necessary for the enrichment method, the DNA of the different specimens was individually barcoded and processed simultaneously, using SeqCap® barcoded adaptors (Roche NimbleGen, USA) (Table 1). Samples were not pooled to avoid concerns of potential *Wolbachia* strain differences. Four different libraries preparations were processed: one Illumina and one PacBio library using DNA enrichment and Illumina and PacBio libraries without enrichment, to determine enrichment efficiency (Table 1). The enrichment method has been described by Lefoulon et al. [36] and it is based on the use of biotinylated probes to capture *Wolbachia* DNA (probes designed by Roche NimbleGen).

For PacBio sequencing (Pacific Biosciences, USA), the Large Enriched Fragment Targeted Sequencing (LEFT-SEQ) method, as previously described, was utilized [36] without the DNA fragmentation step. The PacBio library without enrichment was produced using the SMRTbell® Express Template Prep Kit 2.0 for Low DNA input, using the barcoded Overhang Adapter Kit- 8B (PacBio). The enriched PacBio library was sequenced using 2 SMRT cells with the PacBio RS II system and the library without enrichment used 1 SMRT cell, all on the PacBio Sequel I system.

For Illumina sequencing, a modified protocol was performed, which eliminated the last steps designed for the PacBio library protocol (end repair, ligation of PacBio adaptor and purification) and in which DNA was fragmented using NEBNext® Ultra™ II FS DNA Library Prep Kit (NEB) at 20 °C for 30 min, resulting in DNA fragments with an average size of 350 bp. We used 100 ng of DNA per sample for each capture and used SeqCap barcoded adaptors (Roche Nimblegen) to process simultaneous multiple samples. The Illumina library without enrichment was produced using the NEBNext® Ultra™ II FS DNA Library Prep Kit following the manufacturer’s recommendations (NEB). The enriched Illumina library was sequenced on three independent Ilumina MiSeq runs: one mate-pair 150 bp read and two mate-pair 300 bp reads. The unenriched Illumina library was sequenced with one, single-end, 150 bp NextSeq run. All sequencing was performed at NEB.

### De novo assembly pipeline

Illumina reads were filtered by quality and “cleaned” using the wrapper Trim Galore! (https://www.bioinformatics.babraham.ac.uk/projects/trim_galore/), and then merged with PEAR [62]. PacBio circular consensus sequences (CCS) were generated using SMRT® pipe RS_ReadsOfInsert Protocol (PacBio) with a minimum 3 full passes and minimum predicted accuracy superior at 90. The adaptors were removed by trimming off the first and last 65 bp of the reads, any reads smaller than 20 bp, or reads containing residual adaptor sequences (potential chimeric reads) using seqkt (github.com/lh3/seqtk) (analyses were performed with an in-house shell script).

A first hybrid de novo assembly was done using Unicycler [63]. The potential contigs belonging to *Wolbachia* were detected by nucleotide similarity using blastn [40] and selected (similarity superior at 80%, bitscore superior at 50). The Illumina reads were mapped against this contig selection using Bowtie2 (with the very sensitive settings) [64] and the PacBio reads using ngmlr (with the PacBio preset settings) [65].

A second hybrid de novo assembly was performed using Unicycler with this new selection of reads. Selection of *Wolbachia* contigs was performed using blastn a second time (similarity superior at 80%, bitscore superior at 50) and then verified manually. Assembly statistics were calculated using QUAST [41].

### Comparative genomic analyses and annotation of *w*Apol

The comparative genomic analyses described below included analyses of 10 available complete genomes of *Wolbachia and* 4 draft genomes (Table S8): *w*Mel, *Wolbachia* from *Drosophila melanogaster* (NC_002978), *w*Cau, *Wolbachia* from *Carposina sasakii* (CP041215) and *w*Nfla, *Wolbachia* from *Nomada flava* (LYUW00000000) for supergroup A; *w*Pip, *Wolbachia* from *Culex quinquefasciatus* (NC_010981), *w*Tpre, *Wolbachia* from *Trichogramma pretiosum* (NZ_CM003641), *w*Lug, *Wolbachia* from *Nilaparvata lugens*, *w*Stri (MUIY01000000), *Wolbachia* from *Laodelphax striatella* (LRUH01000000) and *w*VulC, *Wolbachia* from *Armadillidium vulgare* (ALWU00000000) for supergroup B; *w*Ppe, *Wolbachia* from *Pratylenchus penetrans* for supergroup L (NZ_MJMG01000000); *w*Cle, *Wolbachia* from *Cimex lectularius* for supergroup F (NZ_AP013028); *w*Fol*, Wolbachia* from *Folsomia candida* for supergroup E (NZ_CP015510); *w*Bm, *Wolbachia* from *Brugia malayi* (NC_006833) for supergroup D; *w*Ov *Wolbachia* from *Onchocerca volvulus* (NZ_HG810405) and *w*Oo, *Wolbachia* from *Onchocerca ochengi* (NC_018267) from supergroup C.

The Average Nucleotide Identity (ANI) between the *w*Apol draft genomes and available complete genomes of *Wolbachia* was performed using the ANI Calculator [45]. The completeness of the draft genome was studied using BUSCO v3 [43]. BUSCO estimates the completeness of genomes analyzing gene content and comparing to selection of near-universal single-copy orthologue genes (here, 221 genes in common among proteobacteria (proteobacteria_odb9)).

The processed drafts were analyzed using RAST pipeline [42]. Transposable elements were identified: insertion sequences (ISs) using ISSAGA [66] and mobile element and group II introns using RAST pipeline. The phage-like proteins were identified using the RAST pipeline. KEGG Orthology (KO) assignment were generated using KAAS (KEGG Automatic Annotation Server [67]. KAAS assigned orthologue genes by blast comparison against KEGG genes database using BBH (bi-directional best hit) method. The same assignment analysis was performed for the *w*Apol draft genome, the set of 14 complete or draft genomes and 1 supplementary draft genome: *w*Nleu, *Wolbachia* from *Nomada eucophthalma* (LYUV00000000). The assigned KOs were ordered in 165 different KEGG pathways (Table S5). For the biotin operon, the amino-acid sequences were identified with the KAAS assignments of *w*Apol and other studied *Wolbachia* genomes and the complete operons were identified using tblastn and then aligned. The nucleotide sequences were aligned using MAFFT [46], concatenated using Seaview [68] and a phylogenetic analysis was performed with Maximum Likelihood inference inference using IQ-TREE [39].

### Multi-locus phylogeny and Phylogenomics

Two multi-locus phylogenies were performed. The first phylogeny was based on six markers (16S rDNA, *dnaA*, *ftsZ*, *coxA*, *fbpA* and *gatB*), classically used for *Wolbachia* phylogeny [14, 69]. The produced sequences were analyzed with available sequences extracted from 49 *Wolbachia* complete or draft genomes and the addition of sequences from *Wolbachia* from *Zootermopsis angusticollis* and *Zootermopsis nevadensis* (Table S8). A supplementary analysis was based on 13 markers (*groEL*, *fabK*, *nuoG*, *NADH dehydrogenase I subunit F*, *aspS*, *gltA*, *coxA*, *ftsZ*, *wsp*, *orpB*, *nuoD*, isocitrate dehydrogenase gene, TPR domain-containing protein gene) which includes less *Wolbachia* strains (because these markers were rarely used for phylogenetic analyses) but included the only *Wolbachia* known to infect pseudoscorpions, *Wolbachia* from the pseudoscorpion *Cordylochernes scorpioides* [34, 47]*.* This analysis included 14 complete genomes in addition to the *w*Apol draft genome and *w*Csco *Wolbachia* from *Cordylochernes scorpioides* (Table S8).

For phylogenomic analyses, single-copy orthologue genes among a selection of *Wolbachia* genomes were identified using Orthofinder [70]. Two types of phylogenomics studies were performed: one included only 15 complete genomes and one included 36 complete or draft genomes (Table S8). Differences in completeness of draft genomes can be variable and have a negative effect on the robustness of the analyses, and thus the two separate analyses were performed.

The orthologue sequence alignments were generated by MAFFT [46]. For the multi-locus phylogenies, a supermatrix of these six alignments was generated using Seaview [68], and for phylogenomics, the supermatrix was produced by Orthofinder (implemented as functionality). For the later, the poorly aligned positions of the produced orthologue genes alignments were eliminated using Gblocks [71]. The phylogenetic analyses were performed with Maximum Likelihood inference using IQ-TREE [39]. The most appropriate model of evolution was evaluated by Modelfinder [72] (implemented as functionality of IQ-TREE). The robustness of each node was evaluated by a bootstrap test (1000 replicates). The phylogenetic trees were edited by FigTree (https://github.com/rambaut/figtree/) and Inkscape (https://inkscape.org/).

## Supplementary information

**Additional file 1: Figure S1.** Unrooted phylogenetic trees of *Wolbachia* based on 13 markers by Maximum Likelihood. Analysis based on concatenation of groEL, fabK, nuoG, NADH dehydrogenase I subunit F, aspS, gltA, coxA, ftsZ, wsp, orpB, nuoD, isocitrate dehydrogenase gene and the TPR domain-containing protein gene. The total length of the dataset is 6461 bp. The topology was inferred using Maximum Likelihood (ML) inference using IQ-TREE [39]. The Best-fit model, calculated using ModelFinder according to BIC index, was GTR + R3. Nodes are associated with Bootstrap values based on 1000 replicates and only bootstrap value superior to 70 are indicated. The Wolbachia supergroups (A–S) are indicated.

**Additional file 2: Figure S2.** Phylogeny of *Wolbachia* based on the 16S ribosomal RNA gene. Analysis based on alignment of 101 16S rRNA sequences of the total length of 445 bp. The topology was inferred using Maximum Likelihood (ML) inference using IQ-TREE [39]. The Best-fit model, calculated using ModelFinder according to BIC index, was K2P + R2. Nodes are associated with Bootstrap values based on 1000 replicates.

**Additional file 3: Figure S3.** Phylogeny of *Wolbachia* based on the *ftsZ* gene. Analysis based on alignment of 95 ftsZ sequences of the total length of 779 bp. The topology was inferred using Maximum Likelihood (ML) inference using IQ-TREE [39]. The Best-fit model, calculated using ModelFinder according to BIC index, was TIM3 + G4. Nodes are associated with Bootstrap values based on 1000 replicates.

**Additional file 4: Table S1.***Wolbachia* infections in 3 species of pseudoscorpions caught in the Montpellier (France) area determined by PCR amplification.

**Additional file 5: Table S2.** Summary of BUSCOs analyses of *Wolbachia* complete genomes and wApol.

**Additional file 6: Table S3.** List of detected transposable elements.

**Additional file 7: Table S4.** List of phage-like genes detectd by RAST pipeline.

**Additional file 8: Table S5.** Summary of number of genes assigned to 165 different KEGG pathways from *Wolbachia* genomes using KASS.

**Additional file 9: Table S6.** PCR information.

**Additional file 10: Table S7.** NCBI accession of CO1 sequences of pseudoscorpion produced in the study.

**Additional file 11: Table S8.** NCBI accession of *Wolbachia* sequences used in the study and detail of sequences included in molecular analyses. The sequences produced are indicated in bold and blue.

## Data Availability

Data generated are available in GenBank: BioProject PRJNA593570; BioSample SAMN13481355 for *Wolbachia* endosymbiont strain of *Atemnus* sp. Specimen K5 (genome: WQMQ00000000); BioSample SAMN14519337 for *Wolbachia* endosymbiont strain of *Atemnus* sp. Specimen K3 (genome: JAAXCS000000000). The raw data are available in GenBank as Sequence Read Archive (SRA): SRX7550679 to SRX7550680, SRX8097933 to SRX8097936. In addition, a total of 34 sequences were deposited in the GenBank: MN923050 to MN923069, MN931247 to MN931248, MN931689 to MN931700 and MT273088.

## Abbreviations

bp(s): DNA nucleotide base pair(s)
CDS: Coding sequences
G + C: DNA nucleotides Guanine plus Cytosine
contig: continuous DNA segment
IS: DNA insertion element
nt: DNA nucleotide
PCR: polymerase chain reaction
