# Supplementary figures and images for: Pseudoscorpion Wolbachia symbionts: diversity and evidence for a new supergroup S
Source: BMC Microbiol. 2020 Jun 30;20:188. doi: 10.1186/s12866-020-01863-y (PMC7325362; doi:10.1186/s12866-020-01863-y)

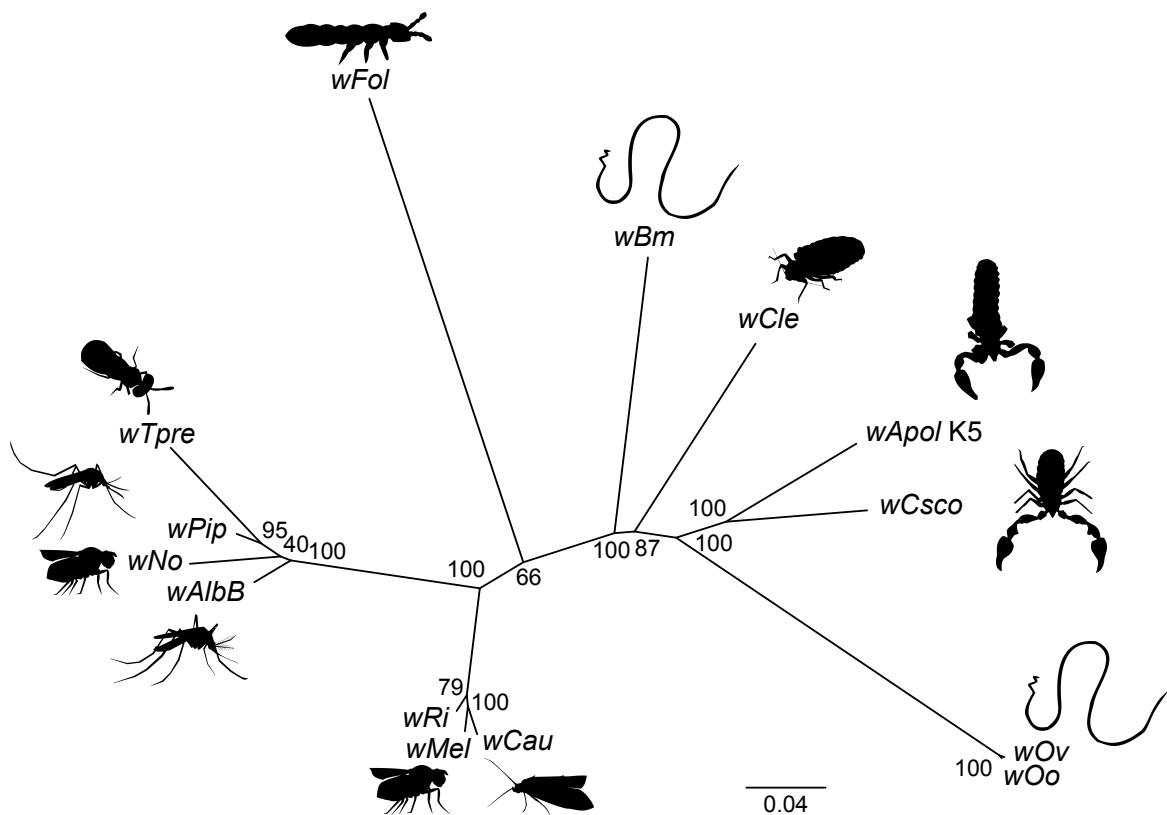

Supplement: Supplementary file 1 — Additional file 1: Figure S1. Unrooted phylogenetic trees of Wolbachia based on 13 markers by Maximum Likelihood. Analysis based on concatenation of groEL, fabK, nuoG, NADH dehydrogenase I subunit F, aspS, gltA, coxA, ftsZ, wsp, orpB, nuoD, isocitrate dehydrogenase gene and the TPR domain-containing protein gene. The total length of the dataset is 6461 bp. The topology was inferred using Maximum Likelihood (ML) inference using IQ-TREE [39]. The Best-fit model, calculated using ModelFinder according to BIC index, was GTR + R3. Nodes are associated with Bootstrap values based on 1000 replicates and only bootstrap value superior to 70 are indicated. The Wolbachia supergroups (A–S) are indicated. [file 12866_2020_1863_MOESM1_ESM.pdf]

Bootstrap: • 100 • >95 • >90 • >80 • >70 • <70

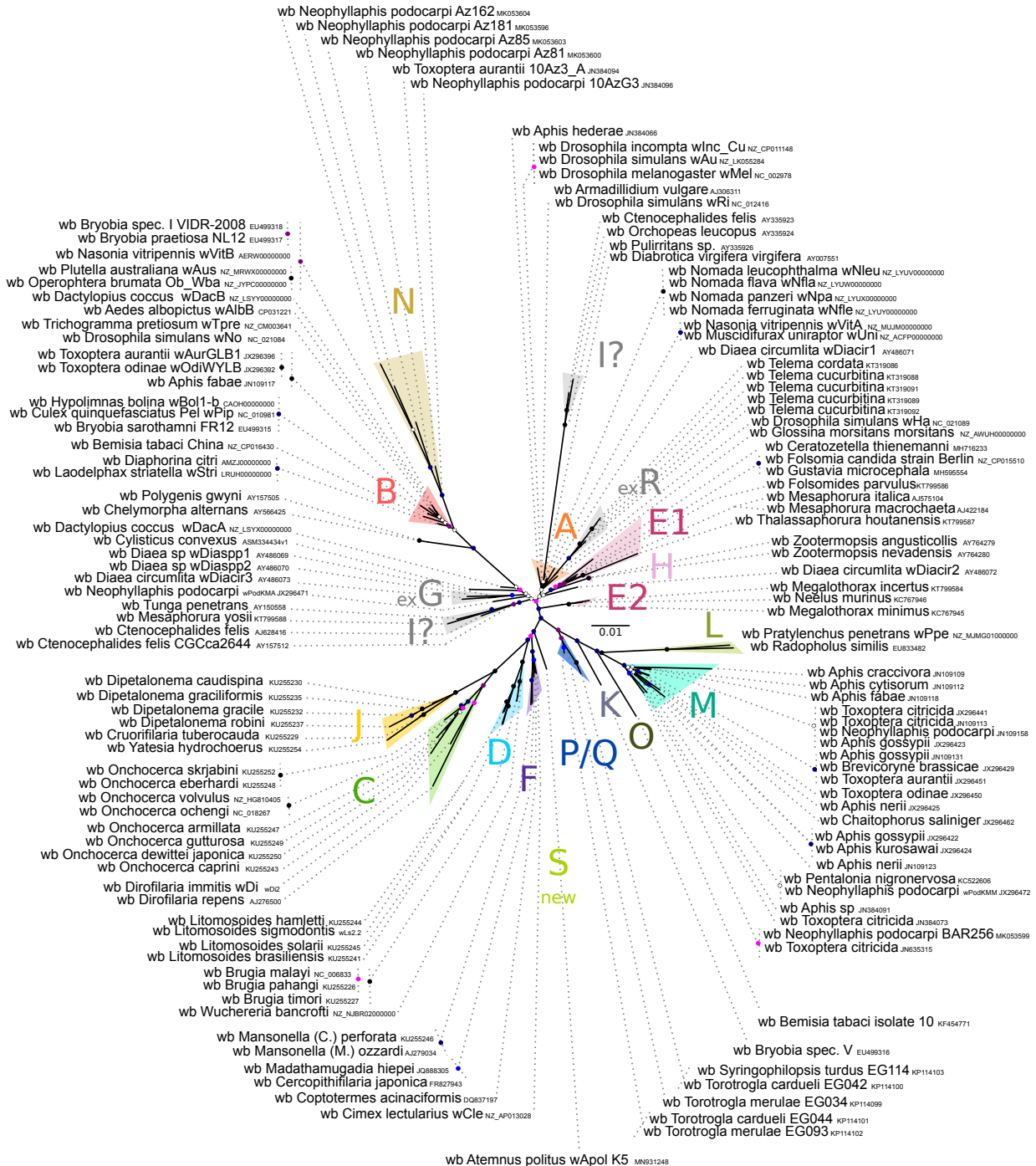

Supplement: Supplementary file 2 — Additional file 2: Figure S2. Phylogeny of Wolbachia based on the 16S ribosomal RNA gene. Analysis based on alignment of 101 16S rRNA sequences of the total length of 445 bp. The topology was inferred using Maximum Likelihood (ML) inference using IQ-TREE [39]. The Best-fit model, calculated using ModelFinder according to BIC index, was K2P + R2. Nodes are associated with Bootstrap values based on 1000 replicates. [file 12866_2020_1863_MOESM2_ESM.pdf]

Bootstrap: ● 100 ●>95 ●>90 ●>80 ●>70 ○<70

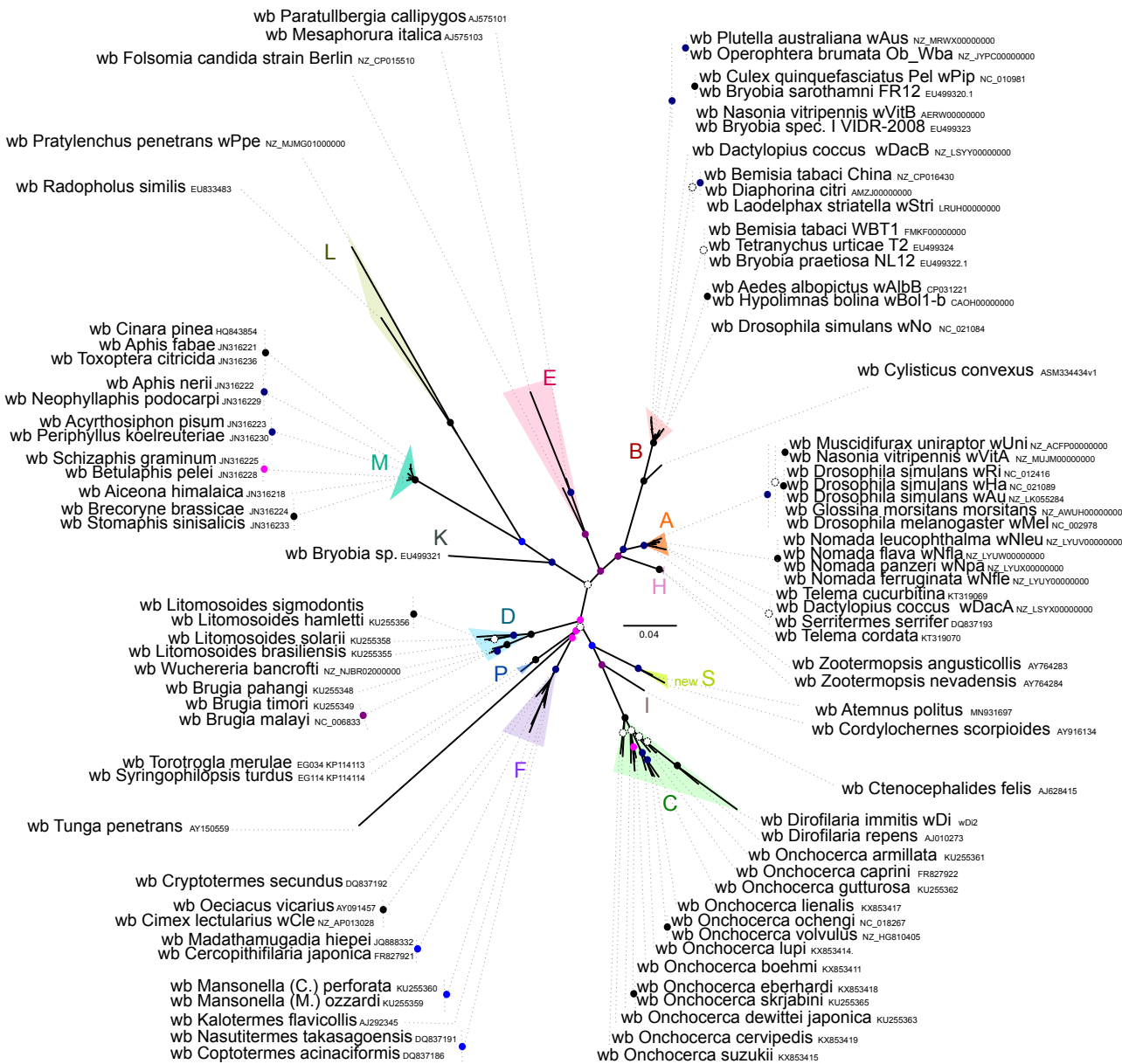

Supplement: Supplementary file 3 — Additional file 3: Figure S3. Phylogeny of Wolbachia based on the ftsZ gene. Analysis based on alignment of 95 ftsZ sequences of the total length of 779 bp. The topology was inferred using Maximum Likelihood (ML) inference using IQ-TREE [39]. The Best-fit model, calculated using ModelFinder according to BIC index, was TIM3 + G4. Nodes are associated with Bootstrap values based on 1000 replicates. [file 12866_2020_1863_MOESM3_ESM.pdf]
